# Assessment of Health-Related Quality of Life after TBI: Comparison of a Disease-Specific (QOLIBRI) with a Generic (SF-36) Instrument

**DOI:** 10.1155/2016/7928014

**Published:** 2016-02-01

**Authors:** Nicole von Steinbuechel, Amra Covic, Suzanne Polinder, Thomas Kohlmann, Ugne Cepulyte, Herbert Poinstingl, Joy Backhaus, Wilbert Bakx, Monika Bullinger, Anne-Lise Christensen, Rita Formisano, Henning Gibbons, Stefan Höfer, Sanna Koskinen, Andrew Maas, Edmund Neugebauer, Jane Powell, Jaana Sarajuuri, Nadine Sasse, Silke Schmidt, Holger Mühlan, Klaus von Wild, George Zitnay, Jean-Luc Truelle

**Affiliations:** ^1^Institute of Medical Psychology and Medical Sociology, University of Medicine Göttingen, Waldweg 37, 37073 Göttingen, Germany; ^2^Department of Public Health, Erasmus Medical College, P.O. Box 2040, 3000 CA Rotterdam, Netherlands; ^3^Institute for Community Medicine, Section of Methods in Community Medicine, Ernst-Moritz-Arndt University, Walther-Rathenau-Straße 48, 17475 Greifswald, Germany; ^4^Hoensbroeck Rehabilitation Centre, Postbus 88, 6430 AB Hoensbroek, Netherlands; ^5^Maastricht University, P.O. Box 616, 6200 MD Maastricht, Netherlands; ^6^Department of Medical Psychology, University Hospital Eppendorf, Martinistraße 52, 20251 Hamburg, Germany; ^7^Centre for Rehabilitation of Brain Injury and Centre for Cognition and Memory, University of Copenhagen, Njalsgade 88, 2300 Copenhagen, Denmark; ^8^IRCCS, Rehabilitation Hospital, Santa Lucia Foundation, Via Ardeatina 306-354, 00179 Rome, Italy; ^9^Department of Psychology, University of Bonn, Kaiser-Karl-Ring 9, 53111 Bonn, Germany; ^10^Department of Medical Psychology, Medical University of Innsbruck, Schöpfstraße 23a, 6020 Innsbruck, Austria; ^11^Institute of Behavioural Sciences, University of Helsinki, Siltavuorenpenger 5 A, P.O. Box 9, 00014 Helsinki, Finland; ^12^Department of Neurosurgery, Antwerp University Hospital and University of Antwerp, Wilrijkstraat 10, Edegem, 2650 Antwerp, Belgium; ^13^IFOM, Private University of Witten/Herdecke, Ostmerheimer Straße 200, 51109 Köln, Germany; ^14^Goldsmiths College, Department of Psychology, New Cross, London SE14 6NW, UK; ^15^Validia Rehabilitation, Department of Clinical Neuropsychology and Psychology, Mannerheimintie 107, 00280 Helsinki, Finland; ^16^Institute for Psychology, Department of Health and Prevention, Ernst-Moritz-Arndt University, 17475 Greifswald, Germany; ^17^Medical Faculty, Westphalian Wilhelms-University, Domagkstraße 3, 48149 Münster, Germany; ^18^Martha Jefferson Association Hospital, 459 Locust Avenue, Charlottesville, VA 22902, USA; ^19^Service of Physical Medicine and Rehabilitation (MPR), Raymond Poincaré Hospital, 104 boulevard Raymond-Poincaré, 92380 Garches, France

## Abstract

Psychosocial, emotional, and physical problems can emerge after traumatic brain injury (TBI), potentially impacting health-related quality of life (HRQoL). Until now, however, neither the discriminatory power of disease-specific (QOLIBRI) and generic (SF-36) HRQoL nor their correlates have been compared in detail. These aspects as well as some psychometric item characteristics were studied in a sample of 795 TBI survivors. The Shannon *H* ^'^ index absolute informativity, as an indicator of an instrument's power to differentiate between individuals within a specific group or health state, was investigated. Psychometric performance of the two instruments was predominantly good, generally higher, and more homogenous for the QOLIBRI than for the SF-36 subscales. Notably, the SF-36 “Role Physical,” “Role Emotional,” and “Social Functioning” subscales showed less satisfactory discriminatory power than all other dimensions or the sum scores of both instruments. The absolute informativity of disease-specific as well as generic HRQoL instruments concerning the different groups defined by different correlates differed significantly. When the focus is on how a certain subscale or sum score differentiates between individuals in one specific dimension/health state, the QOLIBRI can be recommended as the preferable instrument.

## 1. Introduction

After traumatic brain injury (TBI) patients often experience important physical progress within the first six months of recovery. However, cognitive and psychosocial problems continue to persist for the majority of individuals with severe TBI [1]. Patients who have suffered from moderate TBI also report a similar pattern of recovery. Yuh et al. [2] found in their study on individuals after TBI that poorer outcome in physical recovery after three months was associated with abnormalities in MRI, after adjusting for demographic, clinical, and socioeconomic factors. Other imaging studies have described that psychological disorders, such as PTSD, are associated with changes in brain structure [3].

In the past 20 years researchers have reiterated that physical, psychological, and social constraints following TBI present a major challenge to the patients' rehabilitation and their reintegration into society. It has been known in the context of other conditions affecting the brain, such as epilepsy [4], that these factors can exert considerable influence on the HRQoL of patients. Compared to other fields of neurology, research into HRQoL in TBI has only quite recently gained momentum. HRQoL measures capture the patient's own perspective as the best expert of his or her wellbeing and HRQoL. Earlier assumptions that individuals having suffered a TBI would not be able to adequately rate their own HRQoL have more recently been challenged. Now a significant number of studies in TBI successfully employed HRQoL approaches [5].

HRQoL assessment combines multiple domains including physical, psychological (emotional, cognitive), Social, and Daily Life aspects [6]. Measurement of generic HRQoL allows for a comparison across disease states and populations. However, generic tools may not be particularly sensitive for specific aspects and sequelae of a defined disease or health condition. Disease-specific measurement of HRQoL, on the other hand, identifies and targets meaningful, disease- and condition-specific aspects and may therefore be more sensitive to the impacts of the patient's specific health condition.

HRQoL assessment can provide standardized information on recovery patterns and frequency over time, on associations with correlates of disabilities, and on restrictions of wellbeing as viewed from the patient's perspective [6]. Another approach is the use of item banks such as PROMIS [7] and Neuro-QOL [8], which are based on probabilistic measurement models replacing individual instruments and assuring generic and specific evaluation, with the possibility of cross-disease comparability. The application of these systems in TBI is yet still rare [9–11].

It has been evidenced in a number of studies on generic HRQoL that patients afflicted by TBI suffer from a deterioration of HRQoL across all severity degrees [5, 12, 13]. Among the publications using generic HRQoL assessment, the SF-36 health survey is the most widely administered self-rating questionnaire [5]. Overall, studies using the SF-36 have found lower mean scores on all of its eight scales (see below), and on the Mental Component Summary (MCS) and Physical Component Summary (PCS) score for patients with TBI compared with control groups, reflecting poorer health [14, 15].

In disease-specific measurement of HRQoL after TBI the QOLIBRI is the first instrument developed simultaneously in multiple languages. To date there are over 10 studies published reporting on the development and psychometric qualities of the QOLIBRI in TBI populations [6, 16–19] and a number of papers on the application of the QOLIBRI in clinical contexts [20–22]. However, no publication yet has been identified presenting a comprehensive comparison of disease-specific and generic HRQoL after TBI.

A large number of studies have found that sociodemographic characteristics and clinical symptoms are associated with HRQoL, suggesting that the impact of neurological deficits and a changed life situation may lead to poorer HRQoL after TBI [15, 23–27]. Studies also indicate that depression, anxiety, and functional disability have a detrimental effect on HRQoL after TBI [1, 17, 28, 29]. In addition, Stålnacke [25] described that a large proportion of individuals after mild TBI experienced both postconcussion symptoms and psychosocial difficulties, combined with low levels of life satisfaction three years after TBI. Furthermore, elapsed time since injury has been found to also have an impact on generic HRQoL [23]. Results however are not unequivocal; Andelic et al. [30] found no association between HRQoL and functional competence or Employment Status of TBI patients. One paper however briefly examined differences between a disease-specific tool (QOLIBRI) and a generic tool (SF-36) inspecting the associations of the summary scores of the two instruments with the GOSE [17]. Authors concluded that HRQoL assessed with the SF-36 is generally captured also by the QOLIBRI but that there is also additional information available from QOLIBRI scores which was not provided by the SF-36. This study will investigate and compare in detail correlates specific for generic versus disease-specific HRQoL.

Concerning compliance with classical psychometric criteria, validity and reliability of both, the QOLIBRI [16, 17] and SF-36 after TBI [31, 32], have been proven elsewhere. Differences do exist in the QOLIBRI and SF-36 in conceptualization of subjective health, in inclusion of HRQoL domains and items, and in the algorithms used to derive summary scores. Differences in assessing HRQoL in a disease-specific or a generic fashion might have the undesirable effect that the distinct instruments yield different results for similar health states. Furthermore which instrument provides the most solid outcomes with respect to understanding interindividual differences in scale scores is not yet understood. Thus this paper aims at the examination of the discriminative power within a comprehensive sample of TBI patients.

Usually the comparison of HRQoL after TBI determined with a generic [31, 32] and a disease-specific instrument [16, 17] is based on classical psychometric criteria, in which the concepts of reliability, validity, sensitivity, and responsiveness are characterized by the ability to distinguish between “true” different levels of health/HRQoL states [33]. In the study presented here a different approach is applied, the Shannon index *H*′. It originates from the field of information theory but can be applied to any classification, including health classifications and HRQoL systems such as the SF-36, the EQ-5D [34], and the QOLIBRI. The index *H*′ translates the difference between individuals in a given health domain/subscale of an instrument into discriminative power [24, 35]. It also captures a complementary aspect of reliability which cannot be analysed sufficiently when solely investigating consistency coefficients [34].

To gain insight into the diversity of patients the two instruments are able to detect, the current paper analysed the absolute informativity of the QOLIBRI and SF-36 sum scores and subscales. By absolute informativity, we mean the degree to which certain characteristics are distributed among a specific group. If, for example, in a question on educational background with four response options 25% of the sample chooses one option each, high informativity or diversity is detected. However, if 75% of the sample chooses one option and the remaining 25% chooses the other options, informativity of these characteristics with regard to the specific group is low. Furthermore, to be able to investigate whether disease-specific and generic HRQoL are correlated with similar variables, as this may explain variation in discriminative power (*H*′), the QOLIBRI Total score and SF-36 MCS and PCS as outcomes and a number of sociodemographic, psychosocial, and health-related variables as predictors (correlates) were analysed via a stepwise linear regression analysis. (In this paper, in view of terminology differing between disciplines the term correlate is applied instead of the psychometrically correct term predictor.)

## 2. Methods

### 2.1. Participants

The study sample was recruited in six languages (The Netherlands, UK/USA, Finland, France, Germany, and Italy). Data from this patient sample was also published in von Steinbüchel et al. [16, 17]. Around 45% of the patients were in outpatient treatment during participation in our study, and the other did not receive any treatment during the assessment.* Inclusion criteria* entailed the following: ICD-10 diagnosis of TBI, minimum age at injury of 15 years, 17–68 years of age at interview, outpatient treatment (currently not admitted to a ward), and ability to provide informed consent. By the inclusion criterion “outpatient treatment” the exclusion of patient being currently admitted to a ward was intended.* Exclusion criteria were* as follows: a Glasgow Outcome Score Extended (GOSE) [36] below 3, serious current or preinjury psychiatric issues, current severe addiction, diagnosis of a terminal illness, inability to cooperate in the study, and incapability to understand and answer the questions. Patients were asked in a clinical interview and/or records were checked whether they were ever given a psychiatric or addiction diagnosis. If this was the case, their participation was rejected. Ethics clearance was available from each of the recruiting centers.

### 2.2. Medical Information

#### 2.2.1. Clinical Information Checklist

Professionals filled in a checklist which asked for information regarding the patient's clinical background, such as TBI diagnosis, Severity of TBI, current age, age at the time of injury, time since injury, interview mode, and outpatient status. Furthermore, patients' worst GCS score (24 h after injury) was reported as well as the presence of spinal cord injury, current or preinjury psychiatric problems, drug/alcohol dependence, terminal illness, and understanding and communication abilities.

#### 2.2.2. Self-Reported Health-Status List [37]

This questionnaire consisted of a list of 29 symptoms and possible Comorbid Health Conditions with a binary (yes/no) response scale. Three subscales were computed for further analysis: comorbidity (with items on allergies, asthma, thyroid issues, diabetes, back problems, arthritis, high blood pressure, heart disease, angina pectoris, heart attack, use of a pacemaker, bowel inflammation, ulcer, kidney disease, and cancer), sensory/psychosomatic complaints (discomfort with smell, vision, hearing, sleep, headache, nervousness, depression, lack of energy, and lack of physical strength), and musculoskeletal complaints (including problems with movement, paralysis or neurological problems due to TBI, or other than TBI, and amputation of a limb).

#### 2.2.3. Glasgow Coma Scale (GCS) [38]

The Glasgow Coma Scale is a measure of the depth and duration of consciousness impairment and coma. It assesses motor responsiveness, verbal performance, and eye opening and it ultimately classifies brain injury into mild (GCS 13–15), moderate (GCS 9–12), and severe (GCS 3–8). The participants' 24 h postinjury worst GCS score was obtained from medical records.

#### 2.2.4. Glasgow Outcome Scale Extended (GOSE) [36]

This tool determines the grade of disability and recovery concerning the functional status of a patient. For analyses we applied a 3-level categorization of severe disability (GOSE 3-4), Moderate Disability (GOSE 5-6), and good recovery (GOSE 7-8).

#### 2.2.5. Hospital Anxiety and Depression Scale (HADS) [39]

The patient's anxiety and depression levels were self-rated; scores of 8 to 10 represent mild symptoms, 11 to 15 moderate symptoms, and ≥16 severe symptoms [40].

### 2.3. Sociodemographic Data

#### 2.3.1. Sociodemographic and Patient Questionnaire

Participants' Gender, Age, Relationship Status, time since injury, Education, former and current Employment Status, and Living Arrangement were recorded as well as self-perceived Independence, Leisure and Social Activities, and self-perceived health in the past 6 months. Furthermore, the amount of reliance on other people's help (Help Needed) was assessed covering basic personal needs, mobility, daily activities, transportation and organization, and “management of things” in life. Participants were asked to respond on a Likert scale from 1 (“no help needed”) to 5 (“constant help needed”).

### 2.4. Disease-Specific and Generic HRQoL Instruments

#### 2.4.1. Quality of Life after Brain Injury (QOLIBRI) Scale [16, 17]

This disease-specific HRQoL instrument consists of 37 items associated with six subscales including Cognition, Self, Daily Life and Autonomy, Social Relationships, Emotions, and Physical Problems. The first four subscales inquire about the participant's “satisfaction” with different health-related domains of quality of life. The last two subscales ask about how much the participant felt “bothered by” a variety of issues. Answers are given on a 5-point Likert scale that ranges from “not at all,” “slightly,” “moderate,” and “quite” to “very.” The scale means are converted to the 0–100% scale by subtracting 1 from the mean and then multiplying by 25. This produces scale scores with a lowest possible value of 0 (worst possible HRQoL) and a maximum value of 100 (best possible HRQoL). The QOLIBRI provides a disease-specific HRQoL profile over six domains in addition to a total score. Depending on the participant's background, the recruiting centers administered the appropriate language version of the QOLIBRI.

#### 2.4.2. SF-36 Health Survey Version 1 [41]

To capture the patients' subjective health status SF-36 version 1 was administered in all countries but one as a generic outcome measure. In Australia with around 60 patients the SF-36 v2 was applied with subsequently transformed data [16]. It is a multi-item instrument with eight subscales assessing the following domains: Physical Functioning (PF), Role Physical (RP), Bodily Pain (BP), General Health (GH), Vitality (VT), Social Functioning (SF), Role Emotional (RE), and Mental Health (MH). For each domain, a summation of item responses is linearly transformed into a score ranging from 0 to 100. Additionally, a Physical Component Summary (PCS) and a Mental Component Summary (MCS) score are reported. PCS and MCS are calculated by standardizing patients' scores through subtracting US subscale means from each individual's subscale scores. For more details, please look at the manual [41].

### 2.5. Treatment of Missing Values

If less than one-third (33%) of the items were missing, means for each QOLIBRI subscale were calculated, prorated, and expressed as a score from 0 (lowest possible HRQoL measured by the questionnaire), to 100 (best possible HRQoL). A total QOLIBRI score was also calculated as the mean of all individual items, using prorating if necessary. With the missing values in the SF-36 we dealt by overall mean substitution per subscale. The missing values in all other variables (correlates) were treated as missing.

### 2.6. Procedure

Between 2006 and 2008 individuals after TBI were recruited primarily from rehabilitation clinics or from convenience samples in all countries but one. In Germany, patients were consecutively retrieved from community and university hospitals. Patients were identified via archive search and contacted by mail. When interested in participating they were contacted via telephone by the recruiting centers. Upon initial contact, consent was obtained from the participants and arrangements were made for the completion of the questionnaires, considering the physical and cognitive abilities, residence, and mobility constraints of the participants. Those who were able to fill in the questionnaires independently received questionnaires by mail to be returned after completion. When participants needed support, a face-to-face contact was arranged and the GOSE and the inclusion/exclusion criteria were completed in one interview. When postal administration was performed, inclusion/exclusion criteria and the GOSE were completed in a telephone interview. The GCS was then retrieved from the patient record forms.

### 2.7. Data Analysis

Concerning the generic and disease-specific HRQoL instruments (SF-36 and QOLIBRI, resp.) classical psychometric criteria were investigated (mean, standard deviations (SD), skewness of item distribution, floor and ceiling effects per scale, kurtosis, and Cronbach's alpha, as well as convergent and discriminant validity). Probabilistic test theoretical analyses have already been applied for the QOLIBRI [16]. Frequencies, means, SD, and percentages are given for the covariates/correlates of HRQoL. Kolmogorov-Smirnov [42] and Shapiro-Wilk test [43] were used to test if the data were normally distributed. Ceiling and floor effects were defined as answers which fell into the highest or lowest 10% of possible answers per subscale. These effects should be minimal for an instrument to be able to discriminate well.

To be able to investigate whether disease-specific and generic HRQoL share similar correlates, the QOLIBRI Total score and SF-36 MCS and PCS representing the outcomes (predicted values) and a number of sociodemographic, psychosocial, and health-related variables, known from the literature as predictors and correlates, were submitted to a stepwise linear regression analysis (SPSS 22.0). The variables included in the first step were as follows: Years since Injury, Age, Relationship Status, Education, Change of Job, Employment, Living Arrangement, the Degree of Help Needed, Independence, Social Activities, Internet Activities and other Hobbies, Comorbid Health Conditions, Sensory and Psychosomatic and Motor-Skeletal Complaints, GOSE score, and HADS Depression and Anxiety scores. As many of the variables were skewed, we ranked data before subjecting them to regression analyses [44]. The criterion for inclusion of a variable in the final model was that it should independently explain 1% or more of the variance (increase in R2 > 1%) [17].

### 2.8. Shannon Indices

Two different indices will be reported in this paper, *H*′ as the measure for absolute informativity expressed by the number of categories tagged (addressed in detail) and *J*′ as a relative measure that takes into account the maximum informativity which can be reached given a certain number of categories. *H*′ is the absolute informativity captured considering each predictor, whereas *J*′ (evenness) provides the relative informativity. A higher *H*′ means that more information is obtained. The evenness as a relative measure is defined between 0 and 1. A high evenness value indicates a uniform distribution of the response options for a variable. A low evenness index is a sign of a skew distribution.

The Shannon index *H*′ ([45]; see also [33–35]) was calculated for the summary scores and for the separate HRQoL dimensions/subscales. The basic characteristic of Shannon's indices can be explained as follows. In an item with two response categories in which one response category has a very high (or low) endorsement, for example, more than 0.95 (or less than 0.05), the response category transmits very little information because one can predict with more than 95% certainty in what response category the answer will be located [33]. Conversely, in the case of an even distribution, the HRQoL dimension is being most efficiently used, which means that the discriminant ability of the descriptors is maximal. The Shannon index *H*′ combines the number of nonempty categories defined by a system and measures to what extent the information is (empirically) evenly spread over the nonempty categories. As stated above, the higher the index *H*′ is, the more the information is captured by the system. However, the value of the index also depends on the logarithm applied for calculation. Hence, when the logarithm dualis is utilized values are higher than when applying the logarithm naturalis. The logarithm base 2 is defined by log(*p*_*i*_)/log(2), and in our case *p*_*i*_ is the probability of a summary score. We calculated *H*′ by the following formula:

(1) $$\begin{array}{c} H^{\prime }=-\overset{C}{\underset{i=1}{\sum }}p_{i}*\log_{2}p_{i}. \end{array}$$

*C*_*i*_ refers to the number of nonempty categories; *p*_*i*_ is the probability of category *C*_*i*_ and log_2_ is logarithm base 2. The probability *p*_*i*_ of category *C*_*i*_ is estimated by the relative frequencies defined by *n*_*i*_/*N*, where *n*_*i*_ is the frequency of category *C*_*i*_ and *N* is the total number of cases. The Shannon index *H*′ results from summing up the product *p*_*i*_ *∗*log_2_⁡(*p*_*i*_) for all categories. In most cases, especially in information theory, the formula of the Shannon index (SI) is calculated by the natural logarithm and entropy is defined using logarithm base 2. However, in HRQoL research SI has been defined using logarithm base 2 in the formula [34]. In order to assure continuity we decided to define the Shannon index by logarithm base 2. Therefore, we also determined that each score defined a category. After summing up the answers of each person to an individual person score, these scores were used as categories. No grouping was applied. *H*′ max is therefore estimated; it is defined as the maximum value of *H*′ (*H*′max = log_2_(*C*_*i*_)) and indicates the maximum available information. The second index in the context of the Shannon index is *J*′ and is derived from *H*′. *J*′ presents a measure for the spread of the frequencies or categories [34]. The advantage of *J*′, unlike *H*′, is its independence from the number of categories available in the dataset. Statistically, *J*′ quantifies how equally the numbers of categories are distributed. So *J*′, defined by *J*′ = *H*′/*H*′max, indicates how close the number of different categories is. *J*′ ranges between 0 and 1. The more *J*′ deviates from 1, the less the scores are evenly distributed.

Shannon indices were calculated for stratified groups according to differential correlates of disease-specific and generic HRQoL identified through regression analyses for the summary scores and the different subscales of the two instruments (see results section for details). QOLIBRI Total, MCS, the PCS, and the respective subscales are compared with respect to these different correlates of HRQoL. In order to calculate *H*′ for MCS and PCS, we rounded up nonintegers to build categories.

## 3. Results

### 3.1. Patients' Clinical and Sociodemographic Data

Of the 921 enrolled patients 126 were excluded from subsequent analyses as GCS were missing. Thus, for GCS and GOSE 100% of the data were present in the remaining 795 patients; some additional data, however, were missing to different degrees. For demographics concerning Gender and Age, 100% of the data were present and for Living Arrangements, Employment Status, and Relationship Status 93% to 95.6% were present. For Years since Injury 99% of the data was available and 92% for self-reported health-related complaints. HADS Anxiety and Depression scores existed for 99%, for SF-36 subscales for about 91% before imputation. There were less than 5% missing responses per QOLIBRI subscale before imputation. Demographic and clinical characteristics of the final validation study are presented in Table 1. In this TBI sample (*N* = 795), there were a greater number of men (*n* = 537) than women (*n* = 222). Within the age range covered (from 17 to 68 years), three groups of almost equal size were formed (17 to 30 years, 31 to 44 years, and 45 to 68 years).

The mean age was 39 years (SD ± 13.3 years). By GCS criteria, more than half of the sample was severely injured, and for 25% the injury had occurred between 2 to <4 and for 49.6% between four years and 18 years earlier. Less than a quarter of the sample was in full-time employment (which cannot be taken as representative for other TBI samples) and only half in a relationship at the time of the interview. In the different countries, zero (Germany, Australia, and England) to 45% of the patients were still undergoing some kind of therapy or rehabilitation at the time of the assessment. Over half of the TBI survivors were living independently and did not “need help to carry out Daily Life tasks.” Over half of the population reported four or more health-related conditions concerning Comorbidity, Sensory and Psychosomatic, and Motor-Skeletal Complaints. According to the GOSE, the majority (72.4%) of participants were disabled by consequences of their TBI (Severe and Moderate Disability). The mean period of follow-up was 5 years (SD ± 3.9).

### 3.2. Psychometric Analyses of QOLIBRI and the SF-36

As data of the QOLIBRI as well as of the SF-36 (*P* < .001) were not normally distributed, nonparametric tests were applied [16, 17].

In this study additional psychometric criteria, as shown in [16, 17], were investigated in order to compare the metric qualities and the appropriateness of both instruments for administration to individuals after TBI (Table 2).

Concerning item response distributions of the two instruments, for the QOLIBRI subscales no extreme responses (overall ≤20% of extreme responses), for example, no floor or ceiling effects in the responses, were detected, except for subscale Emotion with 24.7% of the answers ranging from 90% to 100% very good to excellent HRQoL (ceiling effects). The ceiling effect for the subscale Emotions had been enhanced by mean imputation. The SF-36, on the other hand, showed both types of effects in most of its subscales: RP resulted in 26.3% responses with floor and 35.7% with ceiling effects, RE had 24.4% floor and 46.2% ceiling effects, and PF, BP, and SF demonstrated 36.8%, 36.4%, and 22.4% ceiling effects and considerably lower floor effects (see Table 2).

Table 2 indicates that psychometric properties of all QOLIBRI and SF-36 subscales, as well for the QOLIBRI Total score and MCS and PCS, were predominantly satisfactory to very good. The two instruments differed in terms of skew, kurtosis, and alpha values. Values for skew as well as for kurtosis all fell within a tolerable range of ±2, which is considered acceptable for a normal univariate distribution [46]. Negative skew for all subscales (Table 2) reflected a pronounced use of the right side of the scales. The latter was confirmed by a significant Kolmogorov-Smirnoff test (*P* < .001). Negative kurtosis denoted a flat distribution across all scales with a more pronounced peak to the right as indicated by negative skew. Subscales RF, RE, and SF of the SF-36 were characterized by floor/ceiling effects, as displayed in Table 2, with more than 50% of the responses for the subscales RP and RE located at the extremes of the answering scales. Cronbach's alpha for both instruments ranged from *α* = .76 to .93 which is considered good to excellent [47].

### 3.3. Correlates of Disease-Specific and Generic HRQoL

To answer the question whether correlates of disease-specific and generic HRQoL do differ, the following variables from the final stepwise regression models for the total score of the QOLIBRI and the summary scores of SF-36 were selected for further analyses: HADS Depression, HADS Anxiety, Help Needed, Social Activities, Comorbidities, Sensory and Psychosomatic Complaints, Motor-Skeletal Complaints, and GOSE categories. With regression analyses in psychometric terms predictors of HRQoL are identified. However, in order to enhance interdisciplinary comprehensibility, we are using the term correlates of HRQoL even though these were identified by multiple regression analyses and not by simple correlation analyses.

The strongest correlates of the QOLIBRI Total score were aspects of the current emotional situation, namely, in descending sequence: HADS Anxiety (27.9%), HADS Depression (10.8%), Functional Outcome (GOSE) (6.3%), Sensory and Psychosomatic Complaints (2.2%), Degree of Help Needed (1.6%), and Social Activities (1%). The strongest correlates for MCS of the SF-36 were HADS Depression (30.5%), HADS Anxiety (9.8%), and Sensory and Psychosomatic Complaints (1.4%), which qualified for further analyses. The strongest correlates for the PCS were Motor-Skeletal Complaints (22%), Comorbidities (6.1%), Degree of Help Needed (5.4%), and Social Activities (3%), as well as Functional Outcome (GOSE) (1.8%), and hence those were suitable for further analysis (Table 3). As expected, the correlates of MCS had mental/psychological aspects in common, and the correlates of PCS captured physical aspects. The correlates of QOLIBRI Total score on the other hand represented a mix of both.

### 3.4. Shannon Indices of Disease-Specific and Generic HRQoL

Here the discriminative power of the two instruments by means of the Shannon index *H*′ was calculated, as was the evenness index *J*′. *J*′ was close to 1 for all Shannon indices *H*′, indicating a uniform distribution of data. However, since the range and directionality of the *J*′ results did not essentially deviate from *H*′, that is, they were comparable, *J*′ (.64 to .97) was not considered for further analyses (data available upon request).

As high Shannon indices indicate high informativity the results clearly showed that SF-36 subscales RP (2.17), RE (1.83), and SF (2.01) had a significantly lower Shannon index/grade of informativity (ranging from 1.83 to 2.17) as well as *H*′ of BP (2.82) in comparison to all other subscales. The Shannon index of the PF was above 3.5, and for MCS and PCS it was above 5. In contrast, all Shannon indices of the QOLIBRI were around 4 (ranging from 3.90 to 4.83) indicating a high discriminative power of all subscales and the total score. As seen in Figure 3, the Shannon indices of PCS and MCS were high. These values can be explained by the great number of categories used in these two summary scales applied for calculation of *H*′.

Next, we inspected the *H*′ index (diversity/absolute informativity) with respect to the two HRQoL instruments for the single correlates in detail.

With respect to Help Needed and Social Activities, the QOLIBRI subscales differentiated well within the correlates, with *H*′ ranging from Physical Problems 3.76 to 4.75 total QOLIBRI. The SF-36 subscales RP, SF, and RE, as expected, did not discriminate well within the correlates (with lowest indices for RE <2); the summary scores MCS and PCS however did differentiate well (with highest index of 5.11 for MCS). Unlike the QOLIBRI subscales, the SF-36 subscales PF and BP differed significantly in their informativity for Help Needed and Social Activities.

Absolute Shannon indices *H*′ of all QOLIBRI subscales indicated that for the three health complaints all differentiated better than did the SF-36 subscales in the whole sample. The Shannon indices *H*′ for the summary scores MCS and PCS, however, were significantly higher than for all other subscales and for the total QOLIBRI score. The subscale Physical Problems (QOLIBRI) transmitted more absolute informativity concerning the single individuals in all three groups of self-reported health complaints than did RP and BP of SF-36. Yet, the informativity of the PF was comparable to the Physical Problems scale of the QOLIBRI.

**Figure 1:**
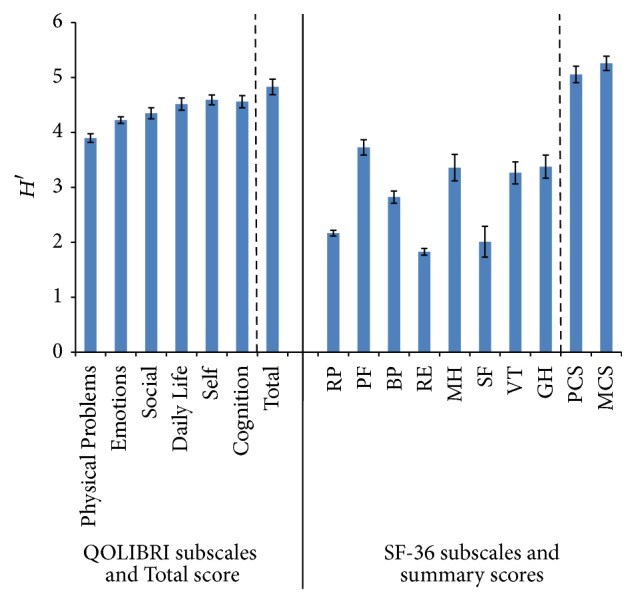
Shannon indices *H*′ (confidence intervals 95%) of all QOLIBRI and SF-36 subscales, QOLIBRI Total score and summary scores.

**Figure 2 fig2:**
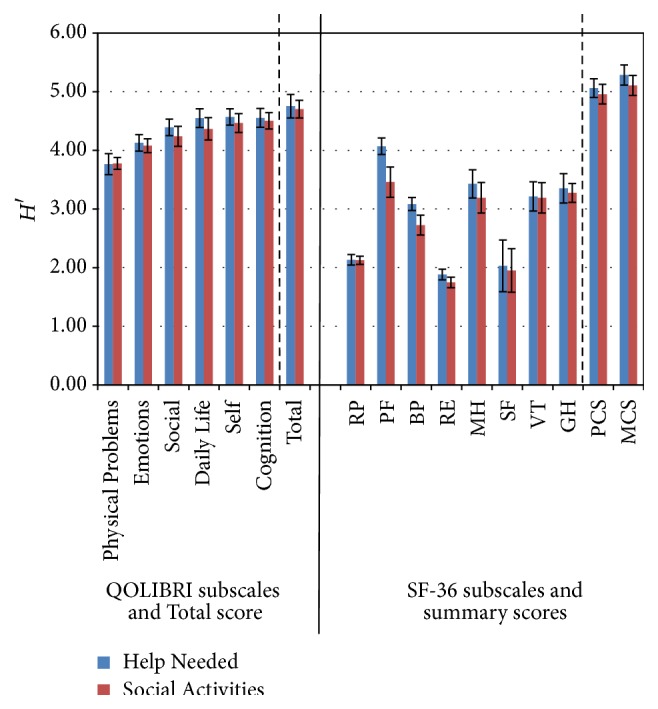
Shannon indices *H*′ (confidence intervals 95%) of all QOLIBRI and SF-36 subscales, QOLIBRI Total score and MCS and PCS for the correlates: Help Needed and Social Activities.

**Figure 3:**
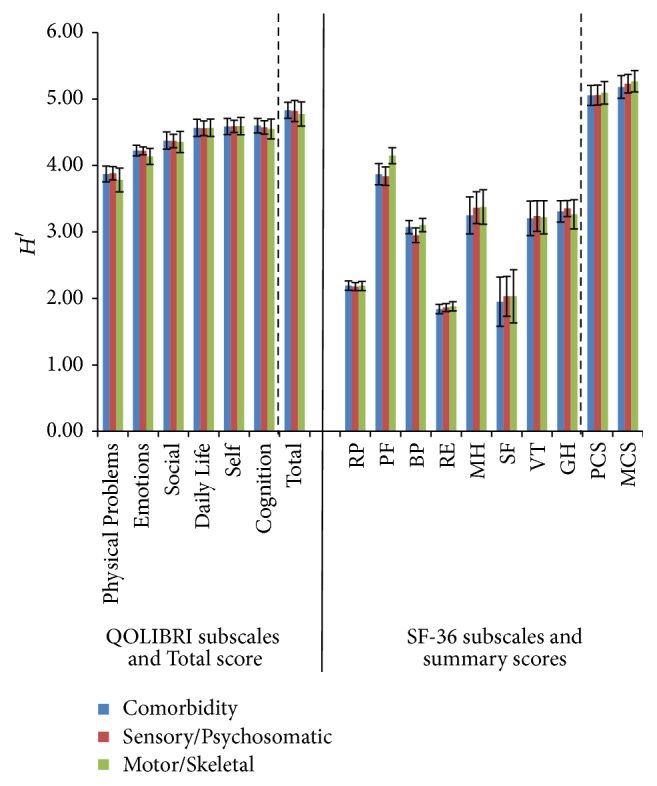
Shannon indices *H*′ (confidence intervals 95%) of all QOLIBRI and SF-36 subscales, QOLIBRI Total score and MCS and PCS for the correlates: Comorbidity, Sensory/Psychosomatic and Motor/Skeletal Complaints.

**Figure 4:**
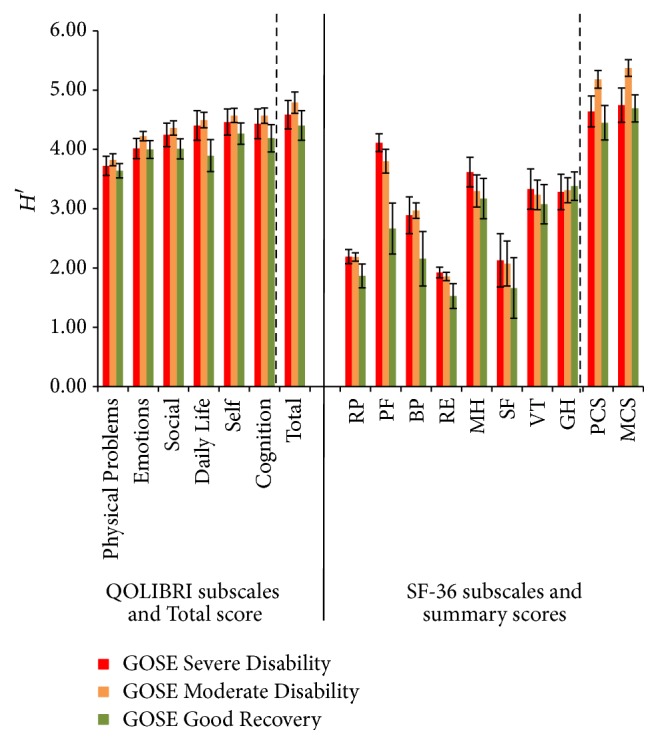
Shannon indices *H*′ (confidence intervals 95%) of all QOLIBRI and SF-36 subscales, QOLIBRI Total score and MCS and PCS for the correlate GOSE scores.

The Shannon indices *H*′ for the RP, RE, and SF reproduced the same pattern as in Figures 1 [Fig fig2]–3 with low informativity concerning individuals belonging to the specific GOSE groups compared to all other scales and summary scores (Figure 4). The QOLIBRI indices, however, ranged from 3.64 (Physical Problems) to 4.57 (Self). Within the QOLIBRI subscales Emotions, Social, Daily Life, Self, and Cognition, the Shannon indices differentiated in the Moderate Disability GOSE category better than in the Good Recovery category, whereby the latter index was lower.

For RP, PF, RE, PCS, and MCS the SF-36 results in a higher informativity for Moderate Disability over Good Recovery. In addition, informativity of MCS and PCS was significantly higher for Moderate Disability than for Severe Disability and significantly differed between Severe and Moderate Disability and between Moderate Disability and Good Recovery.

**Figure 5:**
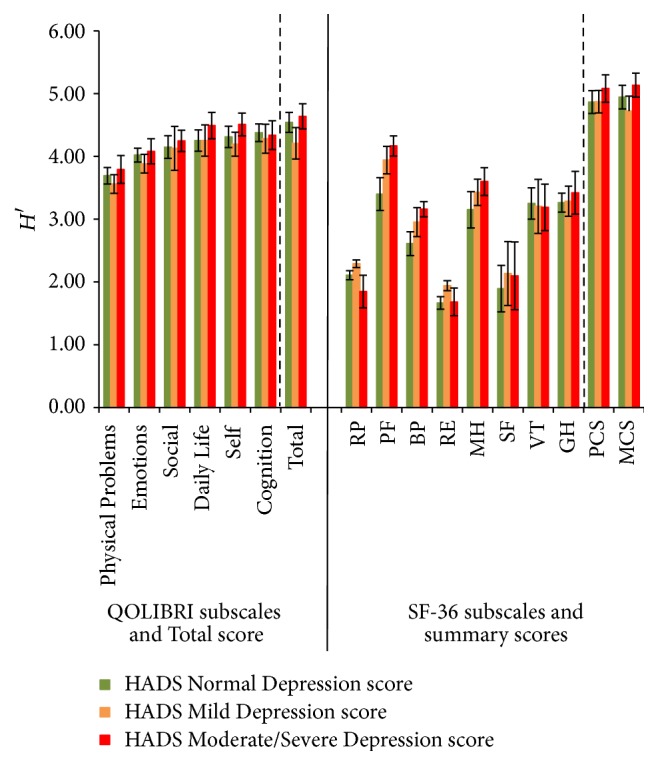
Shannon indices *H*′ (confidence intervals 95%) of all QOLIBRI and SF-36 subscales, QOLIBRI Total score and MCS and PCS for the correlate HADS Depression scores.

The Shannon indices *H*′ for all QOLIBRI scores (ranging from 3.69 for Physical Problems to 4.51 for QOLIBRI Total) and the three GOSE scores were generally higher than for the SF-36 subscales (Figure 4). The Shannon indices did not differ between the score categories per subscale. In the SF-36 subscales, however, we observed considerably more variability. The *H*′ indices range from 1.66 (RE) to 5.14 (MCS). Within the SF-36 subscales, the indices differed significantly for RP with a higher *H*′ for Mild Depression as for Normal and Moderate/Severe Depression. Also, the subscales PF, BP, and RE presented significantly different patterns: HADS Mild and Moderate/Severe Depression scores differed significantly, as did Moderate/Severe and Normal Depression scores but Mild and Normal Depression scores did not. The subscale PF showed the highest *H*′ index for Moderate/Severe Depression scores being, along with the Mild Depression scores, significantly higher than the Normal Depression score *H*′ index. For BP, the Moderate/Severe Depression score was different from the Normal Depression score with the highest *H*′ index. For subscale RE, the Mild Depression score presented with the highest *H*′ index and differentiated significantly between Normal and Moderate/Severe Depression scores. All other indices were comparable to each other (Figure 5).

**Figure 6:**
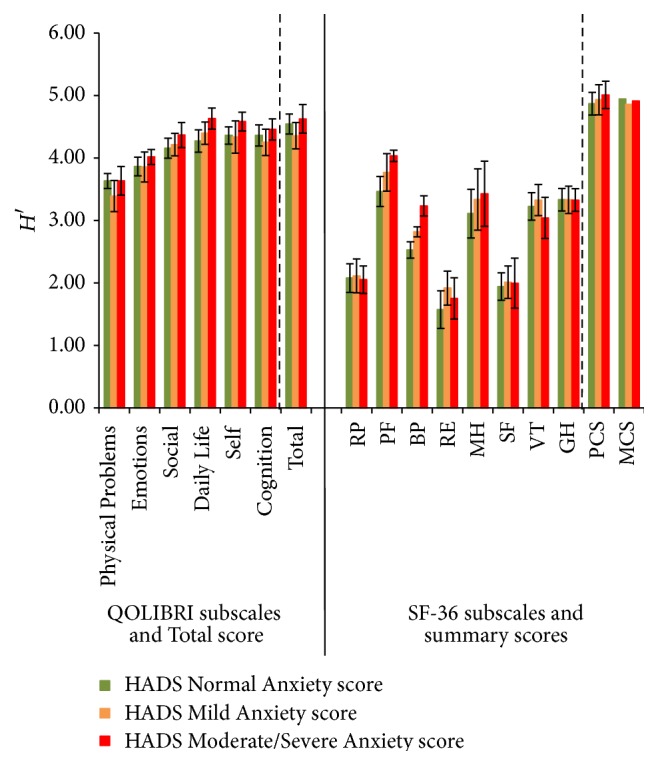
Shannon indices *H*′ (confidence intervals 95%) of all QOLIBRI and SF-36 subscales, QOLIBRI Total score and PCS and MCS for the correlate HADS Anxiety scores.

Also with regard to the HADS three levels of anxiety, the QOLIBRI was characterized by higher indices than the SF-36. The QOLIBRI subscale indices range from 3.39 (Physical Problems) to 4.63 (Daily Life).The Shannon indices *H*′ of the subscales between the score categories were comparable. The subscales of the SF-36 resulted in indices ranging from 1.57 (RE) to 4.04 (PF) and to 5.01 for the PCS. Within the SF-36 subscales, the indices differed for RE, with *H*′ for the Mild Anxiety score being higher than the Normal Anxiety score, PF and BP between Moderate/Severe and the Normal score (Figure 6).

## 4. Discussion and Conclusions

The aim of this paper was a detailed investigation of classical psychometric criteria and the discriminative power/absolute informativity of a disease-specific (QOLIBRI) and a generic (SF-36) HRQoL instrument in the field of TBI.

Psychometric properties of both instruments in a TBI sample were satisfactory to very good, in this and in previous studies. Both instruments are valid and reliable. Item distribution of the QOLIBRI showed no floor effects, but some ceiling effects for the Emotional subscale. For the SF-36 prominent floor (RP, RE) and ceiling effects (RP, PF, BP, RE, and SF) were identified for the majority of subscales. These effects may already indicate that the subscales of the SF-36 might not differentiate sufficiently in a population of TBI survivors.

When comparing the mean values of PCS and MCS with those found in the literature, an overarching effect becomes apparent. In the study of Scholten et al. [48], for example, patients have lower PCS (M = 45, SD = ±10.1) than MCS values (M = 47.2, SD = ±11.6). This pattern is confirmed by Forslund et al. [49], Jacobsson et al. [50], and Grauwmeijer et al. [51]. The latter argued that this finding might be ascribed to the limited awareness of patients after severe TBI who participated in their sample. For our study the results of MCS and PCS are reversed: Values for the PCS (M = 46.70, SD = ±10.1) are higher than for the MCS (M = 43.9, SD = ±11.9). Hawthorne et al. [12] found similar results in mild TBI. These individuals may be less impaired by awareness problems, which subsequently could result in lower MCS values. However, in our study the number of patients who had sustained a severe TBI was twice as large as those who had endured a mild TBI. Thus, these findings have to be analysed in more detail in further studies. A Structural Equation Modeling (SEM) approach incorporating the covariates identified within this study may facilitate understanding of these interactions. In general a measure of awareness can be recommended [52] to scrutinize the relationship between HRQoL ratings in the field of TBI. When comparing the QOLIBRI subscale means and the total score to other studies applying it, mean values are comparable across studies [20–22]. The highest score was determined for the subscale Emotions, followed by Physical Problems. Lower scores were found for Cognition and lowest for the subscale Self. High scores in the subscale Emotions could have been influenced by negative wording, the reversing/recoding of the answers, and the use of mean imputation method. Initially employed to counteract acquiescence response style, empirical evidence exists that reverse coding may actually impair response accuracy [53]. Subsequently, the high scores for the subscale Emotions are not likely to be due to an awareness problem of the patients, especially since the scale Cognition (not reversely scored) presents low values, indicating that an overestimation of cognitive capacities is not present.

Differences observed between the means of the SF-36 and the QOLIBRI may be confounded by different evaluation and/or scoring methods of the instruments. First of all, there are important differences in the applied health/HRQoL state evaluation technique: In the SF-36 response options vary from binary to a six-point Likert scale and weighted summary scale scores. The QOLIBRI in contrast consists of a five-point Likert response and a simple summed up total score.

To investigate the relationship between the instruments applied and the sociodemographic as well as clinical variables, via stepwise regression analyses, correlations of the PCS, MCS (SF-36), and the QOLIBRI Total score were performed. The following correlates of disease-specific and generic HRQoL were identified: for the PCS: Motor/Skeletal Complaints, Comorbidity, Help Needed, Social Activities, GOSE; for MCS: HADS Depression and Anxiety and Sensory/Psychosomatic Complaints. For the QOLIBRI Total score HADS Anxiety and Depression, GOSE, Sensory/Psychosomatic Complaints, Help Needed, and Social Activities correlates were identified. Only a small number of studies use a comparable comprehensive approach for the identification of correlations. Soberg et al. [20] selected correlates for disease-specific HRQoL by multiple regression: the HADS evolved as the most relevant, followed by RPQ, GOSE after 12 months, GOSE after 3 months, and Employment Status. These results correspond partly to ours. We share the authors' assumption that the association between injury severity and HRQoL may dissolve over time, as we also did not find a relationship to GCS in a previous univariate analysis [17]. For over 60% of individuals in our sample the injury had occurred 2 to 18 years prior to participation in the study. In our initial calculations, “time since injury” resulted in significant effects only on three subscales of the questionnaires, for QOLIBRI (Cognition, Self, and Social Relationships) and the SF-36 (RP, BP, and GH). Due to space-constraints, we decided not to focus on time since injury in more detail in this paper. However, the importance of other variables (e.g., psychological and social components) may increase for HRQOL at the later stages of injury.

Since HRQoL is a multifaceted dynamic process affected by many different factors [53] further research with extended correlate models is required. Single correlates such as Functional Recovery, Anxiety/Depression, Race, Gender, and Severity of TBI have been investigated, showing detrimental effects on generic HRQoL in different settings and for varying degrees of severity [27, 51, 54–56]. Unfortunately, in our paper the influence of higher order factors could not be investigated with the analyses applied. A hierarchical linear model will most likely be able to shed light on the complex interplay of variables on different levels.

Subsequently, we discuss our results with respect to differentiating individuals contained in one health state or HRQoL dimension, presented by the Shannon index (*H*′). The SF-36 data implied that the three subscales RF, RP, and RE generally did not have good discriminative power. *H*′ of the remaining SF-36 subscales as well as the two summary scores discriminated well in our TBI population. On the other hand, all QOLIBRI scores displayed high Shannon indices, a result which underlines the necessity of applying specifically tailored instruments to certain diseases. Whereas the generic SF-36 can be used for cross-disease comparisons, the QOLIBRI focuses on consequences important for TBI survivors. Consequently, it should have higher discriminatory power in a specific disease group, which is supported by the results presented within this study. Even though this was not assessed in the present study, but will be in future ones, higher discriminatory power of the QOLIBRI could result in higher sensitivity of this instrument.

As the SF-36 is known to discriminate well between different health/HRQoL states/dimensions in TBI [5], these differences in Shannon indices may initially seem contraintuitive and surprising. Inspecting the response distribution in corresponding individual items and subscales we have however determined pronounced floor and ceiling effects in many of the SF-36 subscales but not for the QOLIBRI. These effects resulted in lower Shannon indices, as frequencies have an impact on the calculation of *H*′. Therefore, these subscales seem to be less sensitive in detecting differences within certain TBI subgroups, when compared to all scores of the QOLIBRI.

TBI outcome is heterogeneous, encompassing a broad spectrum of HRQoL with many problems reported in the physical, emotional, and social functioning domain. In the field of TBI—as in nearly all other medical fields—there is a lack of consensus on preferred HRQoL instruments. To enable straightforward comparisons with other disease groups and with general population norms, it is necessary to measure the consequences of TBI using generic health status measures (as the SF-36 or the EQ-5D) [35]. However, the domains may be not specifically relevant for TBI survivors, as can be deduced from many of our results. There are other important problems that are often identified to be common in TBI, such as cognitive consequences [57], alterations in social relationships [58], limitations to activity and participation [59], changes in the sense of self [60], emotional problems [61], and physical problems. HRQoL in some of these areas typically affected by TBI is not well assessed or not assessed at all by generic HRQoL instruments. In contrast, the QOLIBRI captures multiple of these domains, encompassing especially the psychological (emotional and cognitive), social, and also, to a lesser extent than the SF-36, physical and functional domains [6, 17]. The use of SF-36 in combination with the QOLIBRI is recommended, depending on the focus of a study. For in-group discrimination of patients, requiring a specifically tailored health intervention, the QOLIBRI should be the tool of choice. For comparison and differentiation of individuals between certain health states, health conditions, or HRQoL domains, the SF-36 is an appropriate instrument also. Consensus on preferred methodologies of HRQoL assessment in TBI would facilitate comparability across studies, resulting in improved understanding of recovery and the burden of TBI.

Limitations of the reported study are inherent to the comparison of generic and disease-specific HRQoL instruments. Individual subscales are not directly comparable, as they consist of a different number of subscales and a different number and content of items per subscale, which are not always based on the same theoretical concept. Therefore *H*′ was applied for comparison. Another limitation may be due to the use of version 1 of the SF-36 in this study because of being available in the public domain. Some of the psychometric problems however, especially concerning the physical scales, are described to be solved in version 2 of the instrument [62]. Thus, Shannon indices of the SF-36 v2 subscales remain to be investigated. The currently conducted prospective longitudinal Center-TBI study (https://www.center-tbi.eu/) will allow for this. Furthermore, the influence between the correlates selected and HRQoL could not be investigated in this paper. A Structural Equation Modeling (SEM) approach, as proposed by Williamson et al. [63], could determine whether the correlates are of importance on a precedent level. Interestingly, in recent years many experts refer to TBI as a chronic disease [64], according to whom TBI should be also managed as such. If this chronic nature of TBI is recognized, research could be directed at discovering therapies that may interrupt the disease processes months or even years after the initial event. This paradigm shift would also have an impact on the importance of measuring HRQoL after TBI. Strengths of the study however lie in the attempt to embark in this comparison with a rather new methodology. Shannon indices were calculated in a large number of TBI survivors of all severity levels after applying a comprehensive regression model. In line with our expectations, HRQoL differed depending on GOSE recovery and HADS Depression scores. The QOLIBRI subscales and total score detected more differences between the levels of recovery and depression than the SF-36. In accordance with most studies, lowest HRQoL was observed for severe/moderate depression as well as severe disability. Consistently, HRQoL increased for patients with only Mild Depression and good recovery [30, 65]. Finally, there is a need for longitudinal studies that evaluate possible differential effects over time (such as Center-TBI and Track-TBI). It may well be that correlates are quite different at different time periods after injury.

## 5. Conclusion

Differential correlates were identified for generic and specific HRQoL. In order to better understand HRQoL of patients after TBI, we would like to accentuate a comprehensive assessment of correlates and the use of SEM for future studies. The complex interplay of these factors has to be scrutinized to ameliorate symptoms and tailor interventions for TBI survivors. Based on our novel investigation of the power to discriminate individuals in a health state or in a HRQoL dimension with the Shannon indices *H*′ and *J*′ in comparison with the SF-36, the QOLIBRI is recommended for assessment.

## Figures and Tables

**Table 1:** Demographic and clinical characteristics of the TBI population.

Demographic and clinical variables		Frequency (%)
Age	17–30	271 (34.1%)
	31–44	247 (31.1%)
	45–68	277 (34.8%)

Gender	Male	573 (72.1%)
	Female	222 (27.9%)

Time since injury	<1 year	93 (11.7%)
	1 to <2 years	102 (12.8%)
	2 to <4 years	203 (25.5%)
	4 to 18 years	394 (49.6%)

Relationship Status	Partnered	403 (50.7%)
	Not partnered	303 (38.1%)

Highest Education level	Primary school	42 (5.3%)
	Secondary school and trade or technical certificate	403 (50.7%)
	College diploma or degree	173 (21.8%)
	University degree	88 (11.1%)

Living	Living at home independently	420 (52.8%)
	Living at home supported	245 (30.8%)
	Nursing home	75 (9.4%)

Help Needed	Yes	306 (38.5%)
	No	427 (53.7%)

Leisure activities	Individual activities (internet + hobbies)	425 (53.5%)
	Social activities (socializing + physical activities)	505 (63.5%)
	Hobbies (hobbies + music)	395 (49.7%)

Health Complaints	Comorbidity	440 (55.3%)
	Sensory/Psychosomatic	631 (79.4%)
	Motor/Skeletal	363 (45.7%)

GCS	Severe (GCS < 8)	464 (58.4%)
	Moderate (GCS 8–12)	76 (9.6%)
	Mild (GCS ≥ 13)	255 (32.1%)

Glasgow Outcome Scale-Extended Score	Severe Disability (GOSE 3-4)	143 (17.9%)
	Moderate Disability (GOSE 5-6)	433 (54.5%)
	Good Recovery (GOSE 7-8)	219 (27.6%)

HADS Anxiety score	Normal	496 (62.4%)
	Mild	144 (18.1%)
	Moderate/Severe	152 (19.1%)

HADS Depression score	Normal	537 (67.5%)
	Mild	120 (15.1%)
	Moderate/Severe	132 (16.6%)

**Table 2:** Psychometric characteristics of the QOLIBRI and SF-36 subscales and summary scores.

Descriptive statistics	Mean	(SD)	Floor [%]	Ceiling [%]	Skew	Kurtosis	Cronbach's *α*
*QOLIBRI*							
Physical Problems	67.91	(23.47)	1.4	15.2	.09	.02	.76
Emotions	71.71	(24.69)	2.9	24.5	−.87	.02	.84
Social Relationships	63.65	(22.64)	1.4	14.3	−.41	−.43	.82
Daily Life/Autonomy	66.41	(22.38)	1.3	14.5	−.61	−.11	.87
Self	60.03	(21.96)	2.1	6.2	−.42	−.31	.89
Cognition	61.26	(21.77)	1.0	7.2	−.42	−.34	.89

QOLIBRI Total	64.58	(18.24)	0.4	6.8	−.48	−.04	.82

*SF-36*							
Role Physical	55.13	(38.84)	26.3	35.7	−.21	−1.42	.84
Physical Functioning	76.39	(23.75)	2.3	36.8	−1.15	.71	.93
Bodily Pain	71.28	(26.92)	2.5	36.4	−.61	−.58	.87
Role Emotional	61.01	(39.46)	24.4	46.2	−.46	−1.31	.82
Mental Health	63.90	(20.00)	1.1	8.8	−.46	−.08	.84
Social Functioning	68.01	(25.12)	2.2	22.4	−.59	−.15	.76
Vitality	54.63	(21.51)	4.4	6.5	−.27	−.16	.82
General Heath	63.60	(20.75)	1.1	11.7	−.35	−.33	.76

PCS	46.70	(10.13)	0	0	−.44	−.54	.91

MCS	43.91	(11.94)	0	0	−.40	−.59	.91

**Table 3:** Results of the stepwise regression analysis.

Dependent variable	Proportion of explained variance	Change in *R* ^2^	Significance of change in *R* ^2^
QOLIBRI Total			
HADS Anx.	.278	.279	.000
HADS Dep.	.385	.108	.000
GOSE	.447	.063	.000
Sensory/Psychosom.	.468	.022	.000
Help Needed	.483	.016	.000
Soc. Act.	.492	.010	.001

SF-36-MCS			
HADS Dep.	.303	.305	.000
HADS Anx.	.401	.098	.000
Sensory/Psychsom.	.414	.014	.001

SF-36-PCS			
Motor/Skeletal	.218	.220	.000
Comorbidity	.278	.061	.000
Help Needed	.331	.054	.000
Soc. Act.	.359	.030	.000
GOSE	.376	.018	.000
